# Effect analysis of different methods on radial neck fracture in children

**DOI:** 10.1038/s41598-023-28294-9

**Published:** 2023-01-21

**Authors:** Hailiang Meng, Min Li, Qiang Jie, Yongtao Wu

**Affiliations:** Department of Pediatric OrthopaedicsHonghui Hospital, Xi’an Jiaotong University, Xi’an, 710054 Shaanxi People’s Republic of China

**Keywords:** Bone, Paediatric research

## Abstract

To analyze the curative effect of varying treatment types in 75 pediatric cases of radial neck fracture and explore the prognosis-related factors. Clinical data of 75 children with radial neck fractures treated in our hospital from January 2015 to December 2016 were retrospectively collected. The relationship between age, fracture type, treatment method, X-ray examination after reduction, and prognosis was analyzed. Age was related to prognosis. The excellent and good rate of treatment was 89.25% for children with age ≤ 10 and 57.89% for children over 10 years old. The type of fracture was closely related to the curative effect, 95.0% of O’Brien type I fractures had good curative effects, and the excellent and good rates of O’Brien II type and III type fractures were 87.0 and 66.7%, respectively. According to the type of fracture, the excellent and good rate of patients treated with plaster fixation was the highest (96.42%), but the excellent and good rate was 72.3% in the patients who needed to be reduced by Kirschner wire or elastic intramedullary nail. Although open reduction is superior to closed reduction in imaging evaluation, the excellent and good rate is only 50%.The prognosis of children with radial neck fracture is related to age, type of fracture, and treatment method. In pediatric patients less than 10 years with light, shifted fractures, the excellent and good prognosis rate is higher with less operative intervention. We recommend treating patients with closed reduction and elastic nail fixation according to different fracture types.

## Introduction

Pediatric radial neck fractures (RNF) incidence is low, accounting for 5–10% of elbow fracture injuries^1^. Improper management of neck fractures can affect elbow function and result in serious complications. The management and factors contributing to the different pediatric RNFs are controversial^2^. Children with RNF without displacement or displacement less than 30° can be treated with plaster fixation or closed reduction. For patients with O’Brien’s type II and type III RNF with evident displacement, closed percutaneous reduction, or open reduction, Kirschner’s wire or elastic intramedullary nail fixation was used^3,4^. In the current study, we retrospectively analyzed 75 cases of RNF treated in our department from January 2015 to December 2016 to explore the prognostic factors associated with this injury.

## Materials and methods

### Ethics

This study was approved by the ethics reviewing council of Honghui Hospital, Xi’an Jiaotong University, which abides by the Declaration of Helsinki on Ethical principles for medical research involving human subjects (IRB Approval Number 20190201). Written informed consent was obtained from all participants.

### Clinical data of patients

Clinical data were collected on 75 RNF cases treated in our hospital from January 2015 to December 2016. This information included patient age, sex, causes of trauma, combined injuries, X-ray films, treatment methods, and pain scores in patients who were followed up for one year after treatment. Additionally, the data for complication incidence, elbow flexion, extension, and forearm rotation were collected.

### Treatment strategy

**Figure1:**
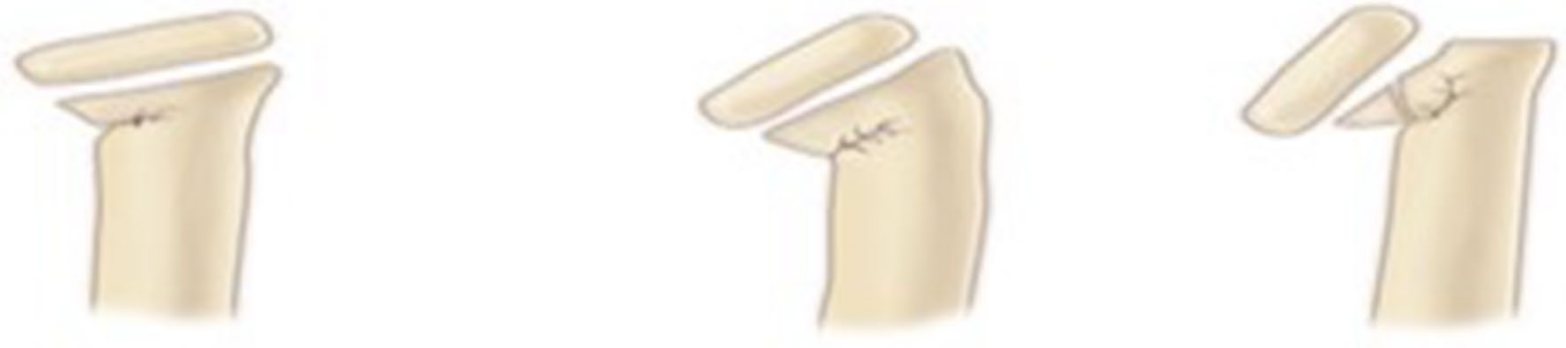
Classification of radial neck fractures in children(O’Brien type).

All patient RNFs were classified using the O’Brien classification criteria^5^ (Fig. 1). Patients with type I RNF were treated by suspending or fixing the limb in the gypsum functional position for 3 weeks. Under general anesthesia, patients with type II RNF were initially treated using manual reduction. If the angle of the radial head was less than 30° after manual reduction, external fixation of the gypsum was performed. Type III RNF patients, as well as type II RNF patients who experienced manual reduction failure patients, were treated using the percutaneous Kirschner prying technique (PKWL)^6^ or the elastic intramedullary nail reduction and fixation technique (CIMP)^7^. If reduction using the Kirschner wire technique failed, the external Kocher approach was chosen for open reduction and internal fixation of the Kirschner wire. External plaster fixation was performed 3 weeks after the operation.

**Figure 2:**
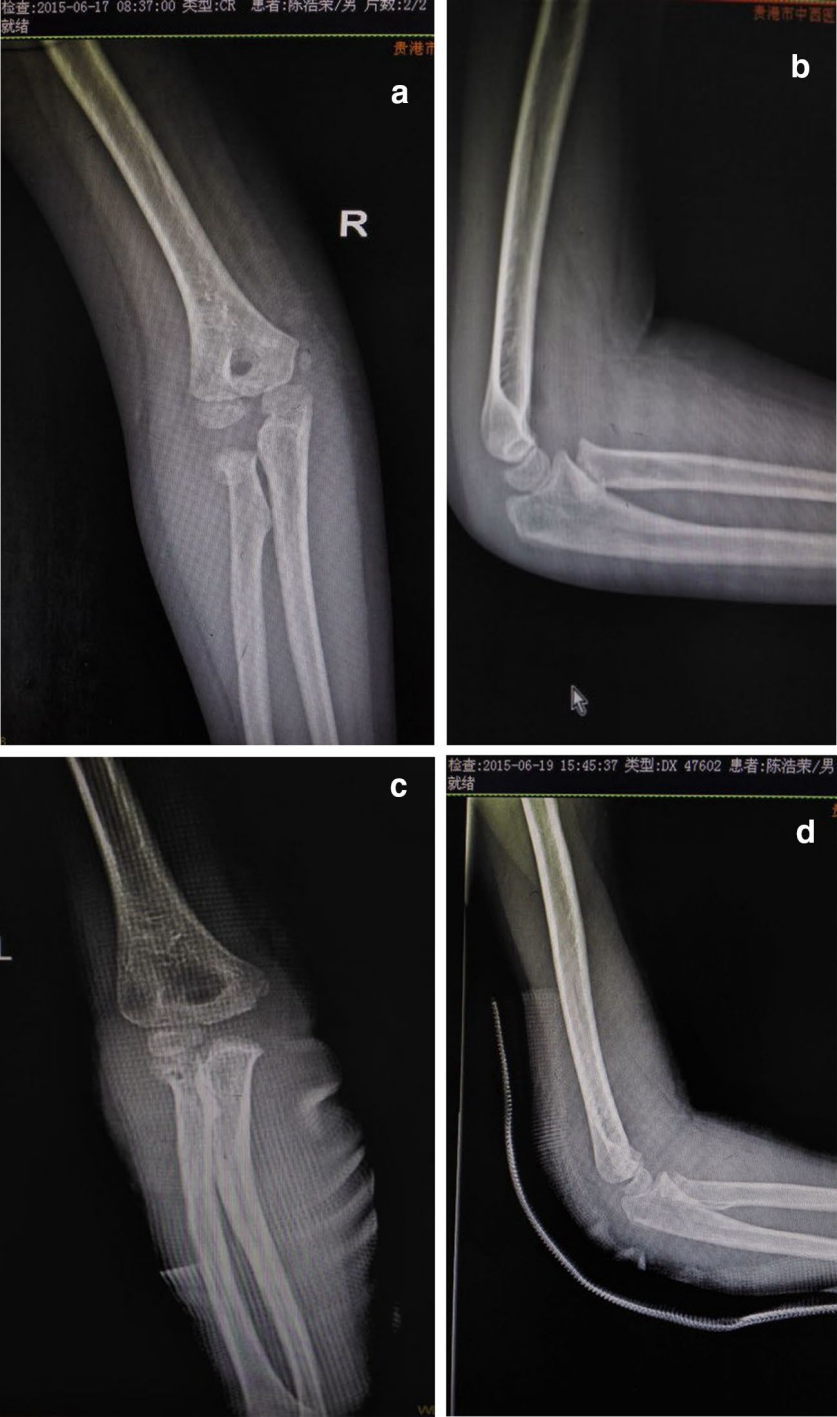
5-year-old boy, right radial neck fracture, type I, plaster fixation (**A)** and (**B**) preoperative , (**C**) and (**D**) postoperative.

**Figure 3:**
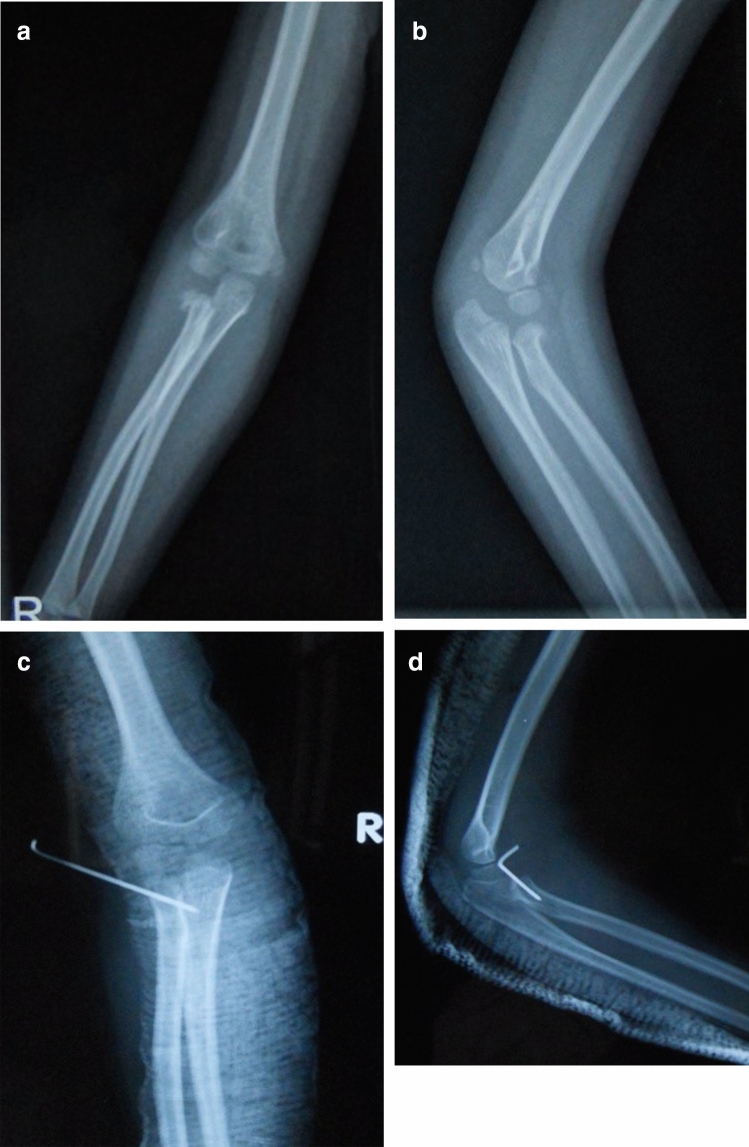
6-year-old girl, right radial neck fracture, type II, PKWL Kirschner wire (**A**) and (**B**) preoperative, (**C**) and (**D**) postoperative.

**Figure 4:**
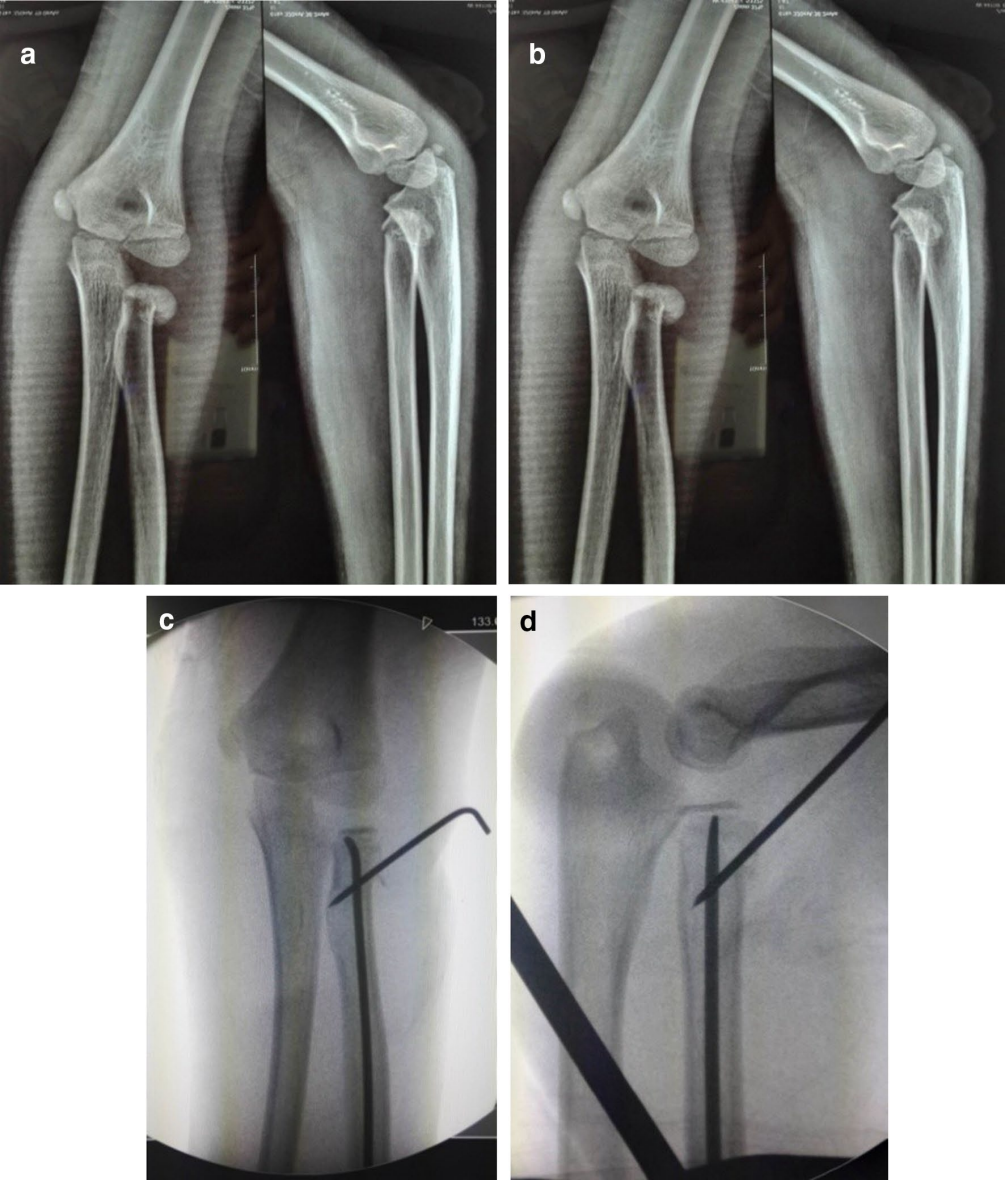
7-year-old girl, left radial neck fracture, type II, PKWL + CIMP (**A**) and (**B**) preoperative, (**C**) and (**D**) postoperative.

**Figure 5:**
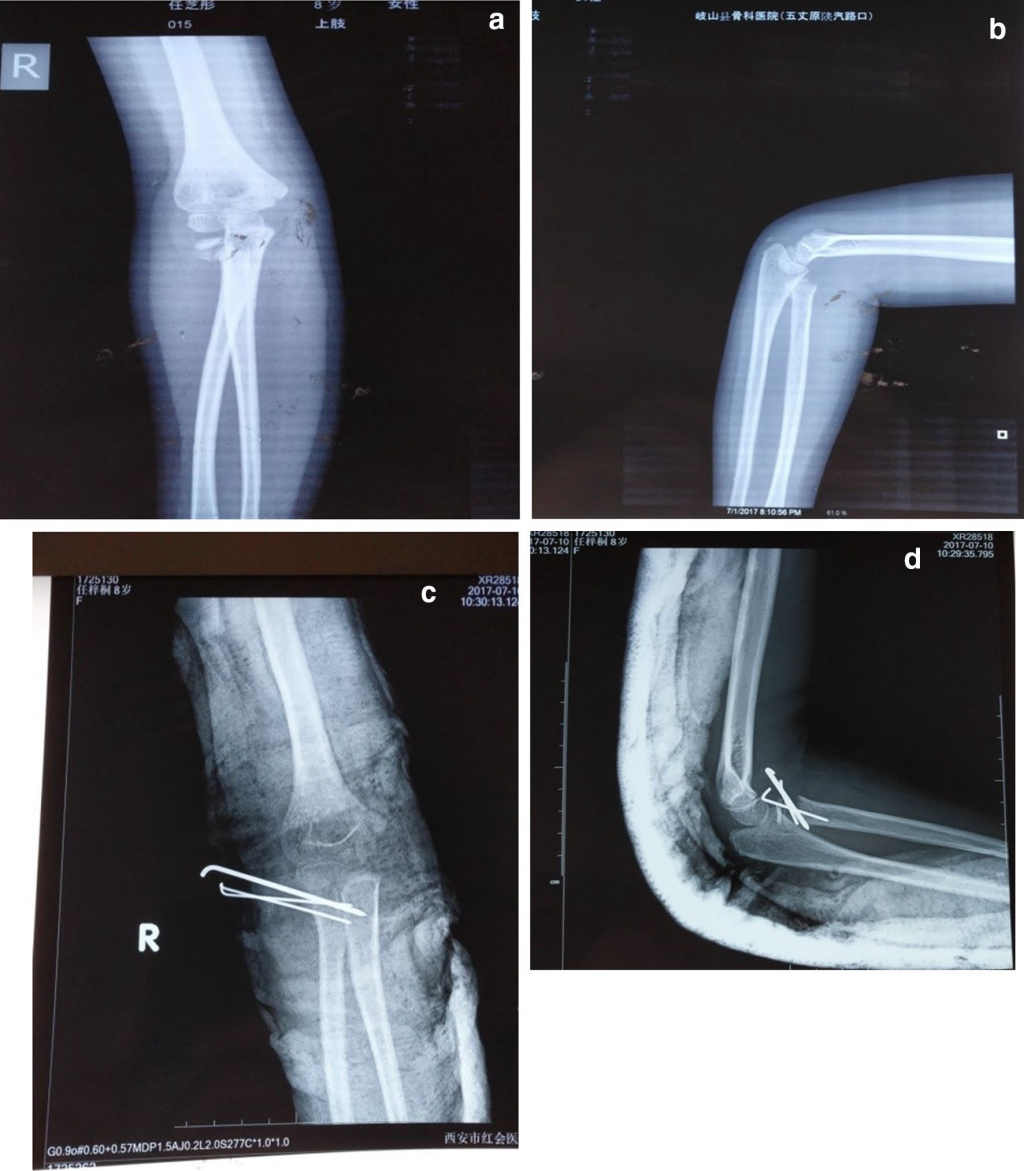
11-year-old girl, right radial neck fracture, type III, treated with open reduction and fixation (**A**) and (**B**) preoperative, (**C**) and (**D**) postoperative.

According to the different treatment methods, the patients were divided into 4 groups, namely Group A (plaster fixation without manual reduction or surgery), Group B (manual reduction and plaster external fixation), Group C (percutaneous prying reduction and internal fixation), and Group D (open reduction and internal fixation) (Figs. 2, 3, 4 and 5 and Table 1).

**Table 1:** Patient groups.

Group	A group	B group	C group		D group
			PKWL + Kirschner wire	PKWL + CIMP	
Number	22	6	11	24	12

### Follow-up plan and evaluation criteria for efficacy

All cases were followed up for more than 12 months. After 12 months of treatment, elbow movement, pain, and deformity were evaluated. Any complications were noted.

#### Imaging evaluation

The effect of plaster fixation was assessed by postoperative X-ray examination of the treatment area according to the Metaizeau reduction criteria^8^ (anatomical reduction, good tilt < 20°, can tilt 20–40°, and poor tilt > 40°).

The function of the elbow joint was evaluated according to the clinical curative effect evaluation method described by Tibone et al.^9^ The elbow joint’s degree of motion, pain, and malformation was divided into four grades (excellent, good, average, and poor). The standard extension and flexion of the elbow joint was 0–145°. 71° pronation and 84° spin backward. Patients with good clinical efficacy were defined as having good prognoses, and those with general and poor clinical efficacy as having a poor prognosis.

### Statistical analysis

All statistical analyses were performed using SPSS version 20. Qualitative data were analyzed by frequency, and the × 2 test compared the two groups. Quantitative data were expressed as (x ± s), and the independent sample t-test was used to compare the two groups. All the factors with significant univariate analysis (*P* < 0. 05) were included in the multivariate non-conditional logistic regression analysis, and the difference was statistically significant (*P* < 0. 05).

## Results

A total of 75 children (33 males and 42 females) with RNFs caused by fall injuries were included in this study. The average age of the participants was 7.9 years (3–4 years), with 56 cases (74.67%) ≤ 10 years and 19 cases (25.33%) > 10 years. The mean follow-up duration was 63.3 months. Of the 75 cases, 27 cases (36%) presented with complicated injuries, including 19 cases of ipsilateral ulnar fracture, 5 cases of medial epicondyle fracture of the humerus, and 3 cases of dislocation of the elbow joint. Of the 75 cases observed, 22 cases were classified as type I, 23 cases as type II, and 30 cases as type III. After diagnosis, the children with type I RNF were treated with plaster external fixation or suspension braking immediately after diagnosis. Children with I type were treated with external plaster fixation or suspension immobilization in the clinic immediately after diagnosis, with a mean time of 1.5 days (0–3d) from injury. II and III were hospitalized, and the average time between surgery and injury was about 3.5 days (2–7 days).

There were 22 cases in Group A, 6 cases in Group B, and 35 cases in Group C. PKWL and Kirschner’s wire fixation were used in 11 cases, and PKWL + CIMP was used in 24 cases (Fig. 4). In Group D, 12 cases were treated using open reduction and Kirschner wire fixation. The most severe displacement cases were in Groups C and D. There were 10 cases in Group D corresponding to type III RNF injury.

### Recovery and complications

All the patients were followed up for an average of 13.5 months (12–24 months). All of the patients’ fractures healed within 3 months. According to the Metaizeau criteria, the excellent and good rates of Groups A, B, C, and D were 75% (21/28), C) (74.29%) (26/35), and 100% (12/12), respectively. According to the clinical evaluation method of elbow function described by Tibone et al. , 39 cases were excellent, 22 were good, 13 were average, and 1 was poor. The incidence of total complications was 25. 33% (19/75) with 12 cases experiencing stiffness (> 20° in any plane), 2 cases of pain, 2 cases of nail tract infection, 1 case of ischemic necrosis, 1 case of tendon injury, and 1 case of back nerve injury.

### Univariate analysis of prognostic results

In 75 cases assessed, 56 cases were aged ≤ 10 years, of which 6 cases had poor prognoses (10.7%) (6/56) (Table 3).Children > 10 years old accounted for 42.10% (8/19) of patients with poor prognosis, and the difference was statistically significant (*P* < 0.05). There was no association between the degree of injury (O’Brien fracture type) and the prognosis (cases analyzed: 1 case of type I, 3 cases of type II, and 10 cases of type III). Among the 75 cases, only 28 cases were treated with gypsum fixation. Of these, only 1 case had a poor prognosis (3.57%, 1/28), but 27.66% (13/47) of children had a poor prognosis in operation group, and the difference was statistically significant (*P* < 0.05). The poor prognosis of patients with percutaneous reduction and fixation was significantly lower (20% (7/35)) than that of patients who underwent open reduction (50.00% (6/12)) (Table 2).

**Table2:** Univariate analysis of poor prognosis in children with radial neck fracture.

	factor	Good prognosis rate(%)	X^2^	*P* value
Age	≥ 10 years old	87.3	5.932	0.013
	> 10 years old	61.1		
Classification of fracture	I type	95.0	2.539	0.016
	II type	87.0		
	III type	66.7		
Therapeutic method	Plaster fixation	100		
	Plaster fixation after reduction	83.3		
	Percutaneous reduction and fixation	72.3	8.034	0.032
	Open reduction	50		

### Multivariate Logistic regression analysis of prognostic results

Age, fracture classification, and treatment methods were taken as independent variables, and the prognosis of the functional score was assessed as the dependent variable. Multivariate logistic regression analysis revealed that RNF patients that were over 10 years (OR = 5.022, 95%CI: 1.160–21.660) were associated with an increased risk for poor prognosis OR = 7.865,95% CI: 0.963–33.894) (Table 3).

**Table 3:** Risk factors of poor prognosis in children with radial neck fracture by logistics regression analysis.

Variable	B	S.E,	Wals	df	Sig	Exp(B)	EXP(B)95%CI
Age	1.610	0.764	4.648	1	0.031	5.021	1.161–21.663
Classification of fracture			0.341	2	0.842		
II type	0.160	1.271	0.017	1	0.889	0.854	0.552–22.636
III type	0.524	0.896	0.337	1	0.559	0.591	0.132–7.885
Therapeutic method			4.41	1	0.026		
Plaster fixation after reduction	1.355	0.784	5.026	1	0.037	5.716	1.337–12.564
Percutaneous reduction and fixation	1.243	0.631	4.791	1	0.042	3.421	1.024–19.225
Open reduction	3.531	0.314	6.542	1	0.014	7.861	0.964–33.893
Constant	6.012	1.511	15.851	1	0	0.002	

## Discussion

RNF in children is mainly caused by angular violence and torsion violence induced by palm bracing after falling^1,10^. Increased external force increases can result in the avulsion fracture of the medial epicondyle of the humerus and fracture of the olecranon and the upper part of the ulna^11^. Small angular and displaced fractures heal without additional treatment; however, displaced fractures often need to be reduced, resulting in the risk of complications caused by improper treatment and impaired elbow joint function^12^. The optimal function of the elbow joint depends on the distal end of the brachial bone, the complete structure of the ruler’s proximal end, and the joint’s stability.

Forearm rotation requires close coordination and compatibility of the ulnar and radial joints, and the proximal axis of rotation is located at the center of the radial neck. Any deviation of the center of the radial head and neck of the radius can change the rotation radians of the radial head. If the head of the radius is shifted to the neck of the radius, the head of the radius will no longer perform smooth circumferential rotation, limiting the movement of pronation and pronation^13^. Several studies have proposed that more than 10% displacement destroys the compatibility of the proximal ulnar radial joint and inhibits radial head rotation around the radius^14^. Therefore, RNFs require sound reduction to avoid deformities. The radial head is covered with cartilage, and blood circulation is supplied from far to near. RNFs are similar to femoral neck fractures, where the blood supply is destroyed, and the radial head is prone to necrosis. Several recent studies have suggested closing manipulation or Kirschner’s wire reduction to treat pediatric RNF and reduce complications^15^.

Many factors, such as age, the severity of the fracture, and the treatment method, are related to treatment outcomes. There are many treatment methods for RNFs in children. Furthermore, the different fracture types require different treatment methods. RNF patients with an O’Brien type I fracture where the radial head angle is less than 30°, direct plaster fixation has been shown to have the best clinical effect. Surgical reduction and internal fixation are required for patients with an angle > 30° or more than 60°. RNF patients with type II fractures should initially be treated using manual reduction. Similar to patients with type III fractures, if the reduction fails, both groups should be treated with surgical reduction and internal fixation. Most RNF patients with type III fractures can be treated by reduction using the percutaneous Kirschner prying reduction technique. Fixation with Kirschner’s wire or elastic intramedullary nail after reduction. Using elastic intramedullary nails reduces the radial head and maintains its position. Additionally, the method has the dual advantage of reduction and fixation, which is beneficial to the early functional exercise of the elbow joint. Few patients with failed percutaneous reduction need open reduction and internal fixation; however, these patients often have O’Brien type III fractures.

Similar to previous studies, this study found that age is related to prognosis following RNF injury^16^. Additionally, the degree of injury (fracture type) is closely related to the prognosis. This study found that the more serious the injury, the more pronounced the fracture displacement, and the lower the excellent and good rate. In our study, the excellent and good rate of closed reduction was high, the excellent and good rate of open reduction was low, and the complications were mainly observed . Plaster fixation had the best curative effect, with an excellent and good rate of 96.43%. Among the patients undergoing percutaneous reduction and fixation, the excellent and good rate was 80%, and the effect of open reduction had the worst clinical effect (50%). Therefore, closed treatment resulted in good results; however, a few patients with severe displacement required open reduction.

Regarding the relationship between the image reduction and the excellent and good rate, the data showed that the orthopedic alignment of a fracture in open reduction was better than that of closed reduction. However, the excellent and good rate of elbow joint function was not significantly correlated with the excellent and good rate of imaging. Therefore, to re-establish the optimal function of the elbow joint according to the Metaizeau restoration standard, an X-ray should meet the standard and not pursue the anatomical reduction of imaging.

RNFs in children are associated with a high complication rate, ranging from 18 to 79%, as reported in the literature .In this study, the incidence of complications is 26.00%, with limited elbow joint movement being the most common complication observed. Additionally, the patients with a nail infection, ischemic necrosis, and interosseous dorsal nerve injury were all treated using the Kirschner wire technique. Therefore, elastic intramedullary nail fixation is superior^17^. For children with obvious RNF displacement, several clinical studies recommend using percutaneous Kirschner wire top prying reduction; however, elastic intramedullary nail fixation is preferred^18–20^.

This study had several limitations. First, although some factors related to the prognosis of pediatric RNF were explored, some indexes, such as rehabilitation exercise, were not assessed. Second, elbow function was only evaluated 12 months after treatment, and some patients may continue to improve their joint function. Last, the number of cases in this group is also small, and further analysis of polycentric data is needed to make robust clinical conclusions.

To sum up, the prognosis of children with RNF is related to age, fracture type, and treatment method. The excellent and good rate of patients who need open reduction is low. Kirschner wire fixation has many complications; however, using the Kirschner wire pushing and top prying technique combined with elastic intramedullary nail fixation is advantageous and results in improved patient outcomes. It is suggested that children with RNFs should be treated according to different fracture types, and those who need operation should be treated using a closed composite elastic nail where possible. Using this method, the alignment of fractures on X-ray can reach the appropriate clinical standard.

## Data Availability

All data should be provided on reasonable request from corresponding author.
