# Author Correction: Effect analysis of different methods on radial neck fracture in children

**DOI:** 10.1038/s41598-023-30277-9

**Published:** 2023-02-21

**Authors:** Hailiang Meng, Min Li, Qiang Jie, Yongtao Wu

**Affiliations:** Department of Pediatric OrthopaedicsHonghui Hospital, Xi’an Jiaotong University, Xi’an, 710054 Shaanxi People’s Republic of China

Correction to: *Scientific Reports* https://doi.org/10.1038/s41598-023-28294-9, published online 21 January 2023

The original version of this Article contained an error in the Funding section.

“This study was supported by a grant from Shaanxi Provincial Key Research and Development Project (CN) (grant no. 2021SF-224) and Xi’an Health Scientific Research Talent Project (CN) (grant no. J202102038). The authors have no conflicts of interest to declare.”

now reads:

“This study was supported by a grant from the Clinical Medical Research Center Projects - Innovation Capability Support Program of Shaanxi Province (2020LCZX-030). The authors have no conflicts of interest to declare.”

The original Article has been corrected.

